# Supplementary material for: Synergistic Anti-Inflammatory Activity of Ginger and Turmeric Extracts in Inhibiting Lipopolysaccharide and Interferon-γ-Induced Proinflammatory Mediators
Source: Molecules. 2022 Jun 16;27(12):3877. doi: 10.3390/molecules27123877 (PMC9229778; doi:10.3390/molecules27123877)
Supplement: Supplementary file 1 [file molecules-27-03877-s001.zip › molecules-1761990-supplementary.pdf]

Table S1. Validation parameters for the method used to quantify ginger and turmeric extracts (n=3 analytical runs).

Compounds	Regression equation	R^2	Limit of detection (µg/mL)	Limit of quantification (µg/mL)	Precision, RSD% Intra-day (n≥3)	Precision, RSD% inter-day (n≥3)
Zingerol	Y=81367x +617.45	0.99	1.59	4.79	0.44	3.32
6-gingerol	Y=60921x + 1312	0.99	0.99	2.97	0.14	0.18
8-gingerol	Y= 51224x + 1547.7	0.99	0.92	2.76	0.19	0.28
10-gingerol	Y = 47288x + 1217.4	0.99	1.59	4.78	0.17	0.49
6-shogaol	Y = 79816x + 74.868	1.00	0.21	0.64	0.16	0.85
8-shogaol	Y = 73090x + 73.626	1.00	0.28	0.85	0.12	0.38
10-shogaol	Y = 74753x + 54.276	1.00	0.19	0.57	0.07	0.25
Curcumin	Y=67047x – 1803.5	0.99	8.88	26.67	0.10	1.27
Desmethoxycurcumin	Y=17556x + 32.161	1.00	0.23	0.68	0.27	0.88
Bisdesmethoxycurcumin	Y= 43107x + 54.821	1.00	0.17	0.51	0.22	1.04

Table S2. Content of bioactive compounds in G and T quantified by HPLC-PDA (n>3) and calculated amount in G-T 5:2 extracts (mg/mL).

Herbal samples	Compounds	Content in G/T (mg/g) determined by HPLC-PDA	Content in G/T at 50 mg/mL (mg/mL)	Content in G-T 5:2 combination at 50 mg/mL (compounds in ginger x 5/7; compounds in turmeric x 2/7) (mg/mL)
G	6-g	69.57±0.16	3.48±0.01	2.48±0.01
	8-g	10.43±0.23	0.52±0.01	0.37±0.01
	10-g	19.62±0.63	0.98±0.03	0.70±0.02
	6-s	7.48±0.19	0.37±0.01	0.27±0.01
	8-s	1.56±0.03	0.08±0.00	0.06±0.00
	10-s	2.30±0.03	0.12±0.00	0.08±0.00
T	C	751.76±101.45	37.59±5.07	10.74±1.45
	B	14.77±3.63	0.74±0.18	0.21±0.05
	D	156.15±26.24	7.81±1.31	2.23±0.09

Table S3. IC_{50} and CI values for compounds combination (combined by their content in G-T 5:2) in inhibiting NO on RAW 264.7 cells.

Individual compounds	NO assay	Compounds combination by their content in GT52	NO assay	
	IC_{50} (μg/mL)		IC_{50} (μg/mL)	CI at IC_{50}
6g	NA*	6g – 6s	91.24±8.50#	6.68
8g	NA	6g - 8s	198.84±10.97#	3.19
10g	NA	6g – 10s	126.99±7.91#	4.69
6s	2.90±1.22	8g – 6s	8.47±0.70	2.85
8s	1.96±1.50	8g – 8s	84.05±4.18	10.69
10s	3.69±0.86	8g – 10s	59.73#	14.26
B	16.14±1.68	10g – 6s	22.06#	5.81
C	5.87±0.12	10g – 8s	1174.99#	146.35
D	8.30±1.78	10g – 10s	24.52#	4.64
		6g - B	43.93±0.67	2.14
		6g - C	16.06±2.41	5.23
		6g - D	9.44±1.42	3.69
		8g - B	27.33±3.75	6.28
		8g - C	12.31±4.41	4.48
		8g - D	4.89±1.07	2.48
		10g - B	17.34±5.38	3.35
		10g - C	10.02±5.28	3.58
		10g - D	11.86±2.86	5.82
		6s- B	12.70±2.09	1.67
		6s - C	16.06±1.12	6.19
		6s - D	9.06±1.16	4.90
		8s- B	12.61±1.64	59.19

		8s - C	13.19±1.91	4.98
		8s - D	6.33±0.81	3.38
		10s - B	40.32±2.50	25.72
		10s - C	10.02±1.95	3.82
		10s - D	9.68±0.92	5.23

*NA: not available; # IC_{50} values were estimated from the measured dose-response curves but not derived from the measured data. STD not available.

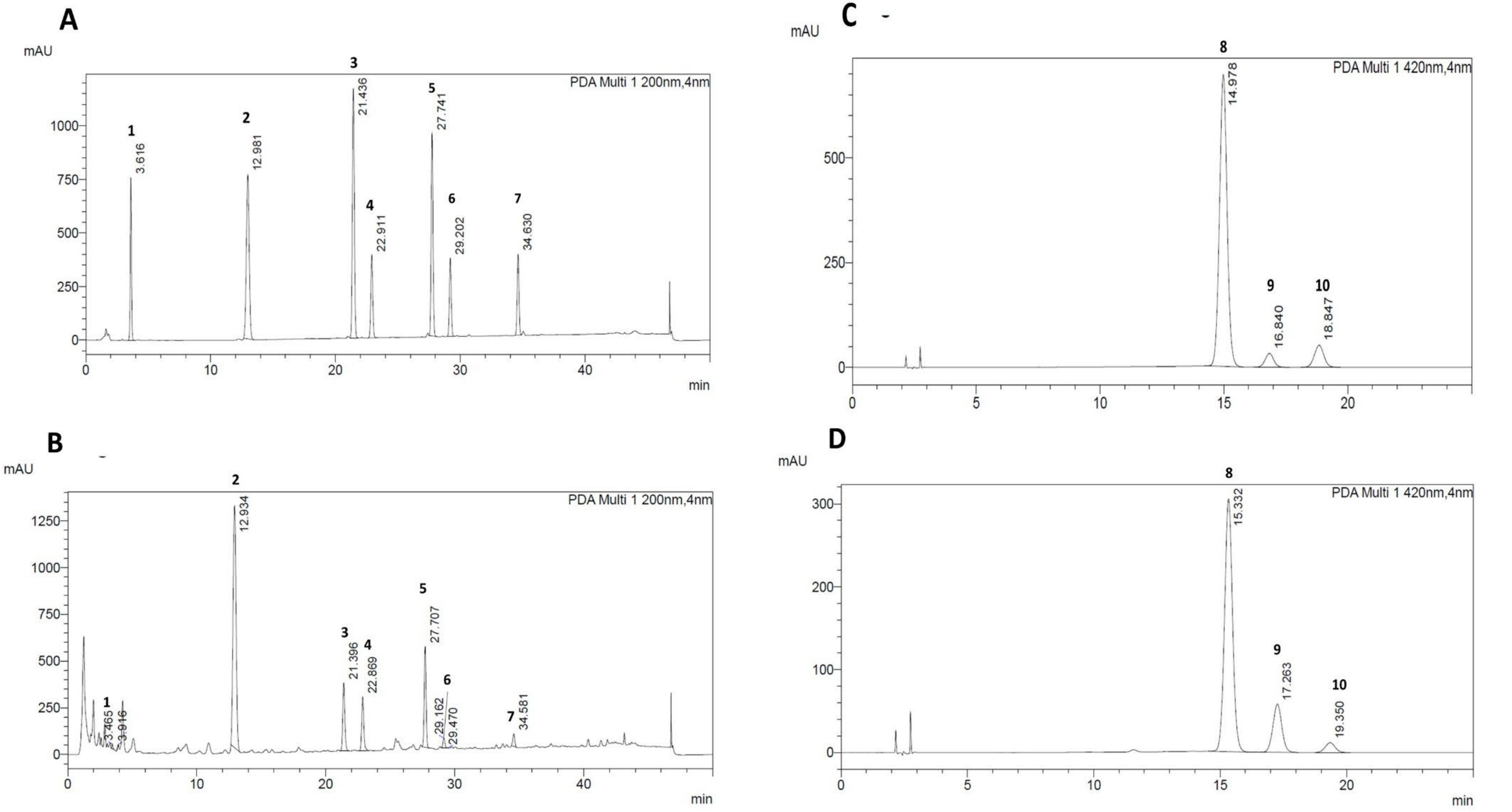

Figure S1. HPLC chromatograms of mixed standards and extracts of ginger (A-B) and turmeric (C-D). 1. Zingerol, 2. 6-gingerol, 3. 8-gingerol, 4. 6-shogaol, 5. 10-gingerol, 6. 8-shogaol, 7. 10-shogaol. 8. curcumin, 9. demethoxycurcumin, 10. bisdemethoxycurcumin.

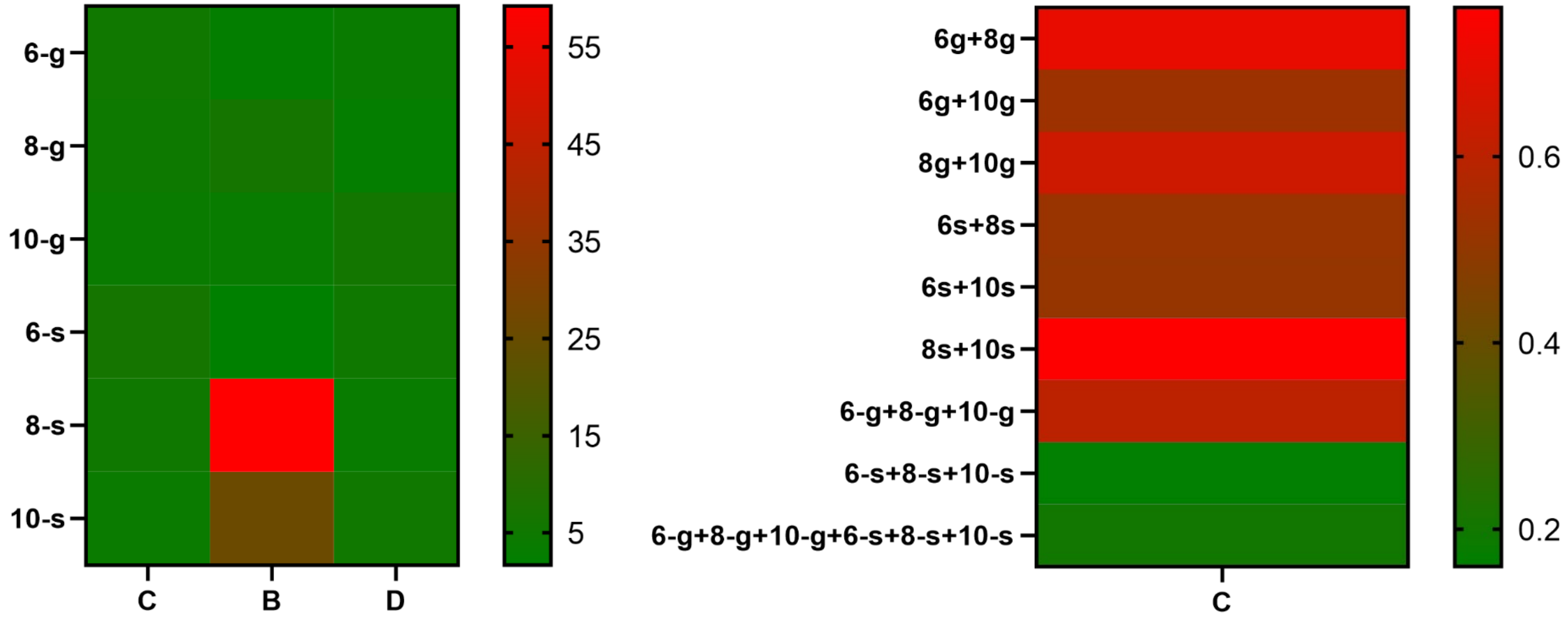

Figure S2. Heatmaps of interaction among tested compounds measured by CI values at IC_{50}. CI>1 refers to antagonism, and CI<1 refers to synergy.
